# Catalytic Reactor-Utilized Ammonia Adsorption, Absorption, and Storage Materials: Mechanism, Nanostructure, and *Ab Initio* Design

**DOI:** 10.1021/acssuschemeng.4c06100

**Published:** 2024-11-19

**Authors:** Aleksandra Zamljen, Blaž Likozar

**Affiliations:** † Department for Catalysis and Chemical Reaction Engineering, National Institute of Chemistry, Hajdrihova 19, 1001 Ljubljana, Slovenia; ‡ Faculty of Chemistry and Chemical Technology, University of Ljubljana, Večna pot 113, 1001 Ljubljana, Slovenia

**Keywords:** ammonia separation, metal halides, kinetic modeling, thermodynamics, computational methods, green energy

## Abstract

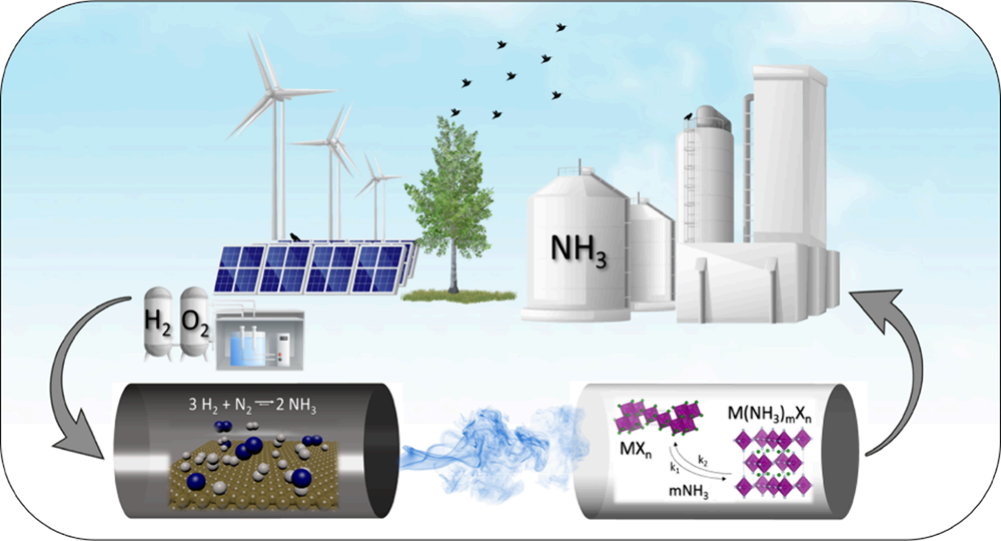

As the world’s technological development shifts toward a sustainable energy future by harnessing renewable energy sources, ammonia is gaining recognition as a complementary green vector to hydrogen. This energy-dense carbon-neutral fuel is capable of overcoming hydrogen’s limitations in terms of storage, distribution, and infrastructure deployment. The biggest challenge to the global use of ammonia as an energy storage medium remains more efficient, readily deployable production of ammonia from abundant, yet intermittent, sources. Green decentralized ammonia production, which refers to the small-scale, localized ammonia production utilizing environmentally sustainable methods, offers a promising approach to overcoming the challenges of traditional ammonia synthesis. The process aims to minimize carbon emissions, increase energy efficiency, and improve accessibility to ammonia in remote regions. Ammonia separation using sorbent materials holds significant potential in green ammonia production, providing a viable alternative to conventional condensation-based separation methods, with particular benefits in improving energy efficiency. This perspective summarizes recent developments in the field of ammonia separation, focusing on newly developed sorbents for the integrated ammonia synthesis–separation process, particularly metal halides that could potentially replace a conventional ammonia condenser. The challenges and potential solutions are also discussed. Moreover, this perspective outlines the mechanism of ammonia absorption into metal halides with its kinetics and thermodynamics. The use of computational methods for the development of new materials is also described, thereby laying the foundations of green ammonia technology.

## Abstract 2

A fundamental modernization of the conventional ammonia synthesis process is crucial for the realization of ammonia as an energy carrier.

## 1 Introduction

The efficient generation, storage, and conversion of renewable energy is extremely important to the sustainable future, addressing critical challenges facing global society, particularly energy cost and security, and climate change. Renewable energy sources are abundant worldwide and cost-effective compared to fossil fuels, therefore contributing to the decarbonization of the energy sector.^1^ Due to intermittency, renewable resources are unreliable and often only available in remote areas, therefore their highly efficient transportation and storage pose the main challenge.^1^ Recent research has focused on cost-effective chemical forms of energy storage, including hydrogen, ammonia, and carbon compounds, which can either be stored for long periods of time at any location or used directly as fuels.^2^

Apart from its use as a fertilizer, which accounts for over 80% of the total production, ammonia is also widely used as a building block for the production of many other products such as explosives, refrigerants, and pharmaceuticals. In addition, ammonia has been extensively studied as a potential energy source for fuel cells, transportation, industry, and power generation,^3,4^ and has also been used for NOx emission control (DeNOx) in automotive applications, which is already commercially available through the use of AdBlue, a high-purity urea solution, utilized in selective catalytic reduction (SCR) systems.^5,6^ Due to its unique properties, ammonia has also been considered as an attractive energy carrier for long-term energy storage. Compared to hydrogen, ammonia is more easily stored as it is in a liquid state at relatively low pressures of about 10 bar at room temperature, or at atmospheric pressure under mild cooling to approximately −33 °C. In contrast, hydrogen storage demands more advanced technologies to maintain its liquid state. Hydrogen must either be compressed to very high pressures, up to 700 bar or more, or cooled to cryogenic temperatures of −253 °C. Both approaches are highly energy-intensive and require more complex infrastructure for safe and efficient storage. Ammonia is a potential hydrogen carrier with a high gravimetric hydrogen capacity of 17.6 wt %, a high energy density of 3 kWh/kg, and its storage and transportation are undemanding, as a reliable infrastructure for the distribution of ammonia is already established.^7,8^ For the efficient implementation of an ammonia-based energy system, ammonia synthesis and storage as well as its utilization are of the main importance.^1,9^

Ammonia is produced industrially by the century-old Haber-Bosch process, with a current global production of approximately 175 million metric tons per year.^10^ The ammonia production cycle is comprised of two main phases: the synthetic mixture production, and the subsequent ammonia synthesis by the Haber-Bosch process.^11^ In the second phase, ammonia synthesis, hydrogen and nitrogen react in the Haber-Bosch reactor under harsh conditions of 200–350 bar and 300–500 °C. An iron-based catalyst is used for the reaction, but due to the low equilibrium single-pass conversion of approximately 15%, the use of gas recycling is required. Prior to the recycle, ammonia product is removed by condensation.^10^ A modern, optimized, and highly efficient methane-fed Haber-Bosch process is still energy-intensive, consuming about 1% of the global energy consumption, highly hydrocarbon-dependent, and accounts for nearly 1.2% of global industrial CO_2_ emissions.^10,12,13^ The challenge for the global use of ammonia as an energy storage medium is therefore to produce ammonia more efficiently, while the method should be adaptable to intermittent energy sources and easily deployable.^14^ Green ammonia synthesis relies heavily on the renewable electricity-powered system, which mainly consists of the water electrolysis subsystem for green hydrogen production, the pressure swing adsorption (PSA) subsystem responsible for nitrogen production, and the Haber-Bosch unit for ammonia synthesis, which could result in zero carbon emissions throughout the ammonia production process, commonly referred to as Power to Ammonia (PtA). The separated ammonia is then stored in a storage tank.^11,15^ By converting renewable energy into ammonia that can be liquefied under moderate pressure, it becomes possible to transport energy from areas with a surplus of affordable renewable energy to where it is limited or expensive.^1,10^

Recently, several innovative approaches have been applied as alternative green pathways for ammonia production. In the field of catalysts, photocatalysis, plasma catalysis, and electrocatalysis have been extensively studied. Another means of improving ammonia synthesis is separation. The conventional Haber-Bosch condensation process, in which the ammonia is separated from the unreacted N_2_ and H_2_ (at −25 to −33 °C and ∼140 bar),^11^ is energy-intensive due to the large temperature swings in the process. Hence, it is only suitable for large-scale, centralized ammonia production. Ammonia synthesis in a small-scale or decentralized plant powered by renewable electricity applied for green hydrogen generation should take place at lower pressures and temperatures. The ammonia yield would be significantly lower compared to the conventional Haber-Bosch process, requiring an alternative NH_3_ separation technology with lower energy consumption than the condensation process.^16^ Since the process using absorption can generate ammonia at a comparable rate but under reduced pressure in comparison to that using condensation, it necessitates a smaller compressor and consumes less energy to operate. Further, synthesis and separation of ammonia can take place within a single vessel, leading to a significantly more flexible process.^11^ Physical condensation utilizes the different boiling points of gases to separate ammonia, while solid absorption utilizes the different solubilities of gases in solid absorbents.^17^ A wide range of solid and liquid sorbents have been used for ammonia separation, such as metal halides, zeolites, borohydrides, metal–organic frameworks (MOFs), covalent organic frameworks (COFs), ionic liquids (ILs), and deep-eutectic solvents (DESs).^16^ For the Haber-Bosch process, it is crucial to find a material that not only complexes a lot of ammonia but does so at higher temperatures. Green ammonia separation materials must be cheap and easy to synthesize, have a high ammonia absorption capacity and, above all, be thermally stable over several absorption/desorption cycles.^18^ The key is to find an optimal balance between thermodynamics, which gives large capacities, and kinetics, enabling large absorption rate.^19^

There have been some reviews on ammonia separation, but most of them focus on the state-of-the-art ILs for liquid ammonia adsorption,^20,21^ or MOFs for solid adsorption,^22^ both of which can effectively separate ammonia only at lower temperatures. The focal point of this review are recent advances in ammonia separation using metal halide materials for solid absorption, which could be used at higher temperatures and thus implemented in the ammonia synthesis process. First, the porous materials are briefly described, followed by a detailed description of the metal halides, their structure, and the mechanism of ammonia separation. Kinetic modeling and thermodynamics of metal halides are further described, as well as computational methods for the development of new materials. Finally, an outlook on the future is provided to give perspective to researchers in the field of ammonia separation.

## 2 Ammonia Separation Materials: Adsorption versus Absorption

Both adsorption and absorption are equilibrium phenomena, i.e. they reach a stable state under specific experimental conditions. Therefore, when measuring adsorption and desorption, it is crucial to specify the conditions accurately, particularly temperature and pressure, which is not always the case in the literature (see Tables 1 and 2). Although both involve equilibrium state, adsorption and absorption differ in terms of the processes and conditions that govern their equilibrium states. The term absorption is used when a target species is taken up by reaction into a solid, in the case of metal halides into a crystal, where equilibrium is reached when the rate of absorption equals the rate of desorption or when the concentration of the absorbed substance in the medium reaches a constant value. Adsorption is the uptake onto the surface of a solid, where the equilibrium is determined by the balance between adsorption and desorption rate.^23−25^ The solid is preferably microporous so that a larger surface area is available. While absorption is selective and can occur at temperatures higher than ambient, adsorption is less selective and can decrease more rapidly with increasing temperature. When both absorption/adsorption and desorption are considered, the term sorption is used.^26^ During absorption, the absorption bed binds ammonia until the ammonia concentration of the gas exiting the bed suddenly increases or, as often referred, breaks through. The breakthrough time is a reliable measure of the capacity of the bed.^27^ The most important properties of absorption materials are high absorption capacity, high absorption/desorption rate, and cyclic stability. These essential properties are defined by some critical factors, in particular surface area, porosity, and affinity to ammonia.^28^ The cyclic absorption/desorption process is a multiobjective optimization problem that requires a thorough investigation because of trade-offs between absorption capacity, absorber temperature, pressure in the synthesis loop, recycle rate, and the absorber size.^29^

**Table 1:** NH_3_ Absorption Capacities, BET Surface Area, Regeneration Temperature, and Stability of Representative Bulk Metal Halides Reported in Literature

material	*T* [°C]	*P*_abs_ [bar] (*P*_parc.NH3_ [bar])	absorption capacity [mg_NH3_/g_sorbent_]	absorption capacity [mol_NH3_/mol_sorbent_]	BET [m^2^/g]	regeneration	stability	ref
MgCl_2_	25	1	1001.3	5.598	3	/	Crystal structure retained up to 600 °C under vacuum; in air atmosphere MgO is produced above 500 °C	(47)
MgCl_2_	150	4 (0.66)	7.2	0.040	/	/	/	(19)
MgCl_2_	200	4 (0.66)	1.7	0.010	/	/	/	(19)
MgCl_2_	300	2 (0.33)	7.7	0.043	/	450 °C, 30 min	BT time drops significantly within 5 cycles	(50)
MgCl_2_	25	(0.8)	931.6	5.208	/	25 °C; vacuum	/	(49)
MgBr_2_	150	4 (0.66)	7.4	0.080	/	/	/	(19)
CaCl_2_	25	(0.8)	646	4.210	/	25 °C	/	(49)
CaCl_2_	25	/	380	2.476	13	/	/	(51)
CaCl_2_	150	4 (0.66)	1.1	0.007	/	/	/	(19)
CaBr_2_	25	(0.8)	499.8	5.866	/	25 °C	/	(49)
CaBr_2_	150	4 (0.66)	4.9	0.058	/	/	/	(19)
SrCl_2_	22	3	798.49	7.433	/	/	/	(52)
SrCl_2_	150	4 (0.66)	0.16	0.001	/	/	/	(19)
SrBr_2_	25	(0.8)	521.9	7.583	/	25 °C	/	(49)
SrBr_2_	150	4 (0.66)	2.4	0.035	/	/	/	(19)
MnCl_2_	25	/	580	4.286	13	/	/	(51)
CuCl_2_	25	/	670	5.289	15	/	/	(51)
CuCl_2_	25	/	660	5.210	15	200 °C	Loses capacity in the 2nd cycle, then stable in next 9 cycles	(48)
CuBr_2_	25	/	460	6.033	12	200 °C	Loses capacity in the 2nd cycle, then stable in next 9 cycles	(48)
CuI	25	/	300	3.355	5	200 °C	Stable over 10 cycles	(48)

**Table 2:** NH_3_ Sorption Capacities, BET Surface Area, Regeneration Temperature, and Stability of Representative Bulk Metal Halides Reported in Literature

material	*T* [°C]	*P*_abs_ [bar] (*P*_parc.NH3_ [bar])	sorption capacity [mg_NH3_/g_sorbent_]	BET [m^2^/g]	wt % salt	regeneration	stability	ref
MgCl_2_/silica	25	2.8	200	/	33	400 °C	/	(29)
MgCl_2_/silica	25	/	560	13	/	/	/	(51)
MgCl_2_/silica	200	2.8	60	/	33	400 °C	/	(29)
MgCl_2_/silica	150	4 (0.66)	69	/	40	/	/	(19)
MgBr_2_/silica	150	4 (0.66)	62	/	40	/	/	(19)
CaCl_2_/silica	25	/	430	12	/	/	/	(51)
CaCl_2_/silica	150	4 (0.66)	33	/	40	/	/	(19)
CaBr_2_/silica	150	4 (0.66)	61	399	40	/	/	(19)
SrCl_2_/silica	150	4 (0.66)	7.5	/	40	/	/	(19)
SrBr_2_/silica	150	4 (0.66)	20	/	40	/	/	(19)
CuCl_2_/silica	25	/	640	42	/	/	/	(51)
MnCl_2_/silica	25	/	610	40	/	/	/	(51)
silica	150	4 (0.66)	10	540	0	/	/	(19)
ZnCl_2_/MCM-41	25	/	150	396	50	/	/	(64)
Cu(NO_3_)_2_/MCM-41	25	/	122.7	614	30	/	/	(64)
Zn(NO_3_)_2_/MCM-41	25	/	114	568	30	/	/	(64)
MCM-41	25	/	34	930	0	/	/	(64)
MgCl_2_/HMSS	30	(0.8)	340	149	80	/	/	(57)
HMSS	30	(0.8)	72	1594	0	/	/	(57)
MgCl_2_/alumina	25	(0.06)	58.6	/	5.9	450 °C	BT curves reproducible at least 6 cycles	(41)
MgCl_2_/alumina	150	2 (0.33)	18	0.5	6	450 °C, 30 min	5% reduction in capacity up to 40th cycle	(50)
MgCl_2_/alumina	300	(0.06)	11.6	/	5.9	450 °C	BT curves reproducible at least 6 cycles	(41)
CaBr_2_/alumina	150	4 (0.66)	2.6	1	40	/	/	(19)
CaCl_2_/alumina	25	(0.06)	32.7	/	5.7	450 °C	BT curves reproducible at least 6 cycles	(41)
CaCl_2_/alumina	300	(0.06)	6.5	/	5.7	450 °C	BT curves reproducible at least 6 cycles	(41)
BaCl_2_/alumina	25	(0.06)	15.7	/	5.4	450 °C	BT curves reproducible at least 6 cycles	(41)
BaCl_2_/alumina	300	(0.06)	2.8	/	5.4	450 °C	BT curves reproducible at least 6 cycles	(41)
CaCl_2_/γ-alumina	25	5–8	225	/	21.5	/	/	(56)
alumina	25	(0.06)	13.2	220	0	450 °C	BT curves reproducible at least 6 cycles	(41)
alumina	150	2 (0.33)	8.5	155	0	450 °C, 30 min	Gradual decrease in capacity	(50)
alumina	300	(0.06)	3.3	220	0	450 °C	BT curves reproducible at least 6 cycles	(41)
CaBr_2_/zeolite Y	150	4 (0.66)	34	541	40	/	/	(19)
CaBr_2_/kaolinite	150	4 (0.66)	5.2	<1	40	/	/	(19)
CaBr_2_/diatomaceous earth	150	4 (0.66)	1.7	2	40	/	/	(19)
CaCl_2_/vermiculite	25	5–8	685	/	63.5	/	/	(56)
MgCl_2_/GNA	25	1	810.9	97	80	200 °C	stable at least 3 cycles	(47)
MgCl_2_/Gt	25	1	795.6	5	80	200 °C	/	(47)
MgCl_2_/AC	40	7 (0.35)	146.2	1283	4	25–200 °C, incomplete	maintained high capacity over 15 cycles	(65)
CaCl_2_ + ENG	15	/	736.8	/	/	/	/	(66)
CaCl_2_ + ENG	80	12	356	/	80	/	/	(67)
SrCl_2_/rGO	22	3	722.3	14	80	pressure drop (3 bar to high vacuum)	stable capacity over 20 cycles	(54)
rGO	22	3	90.1	39	0	/	/	(54)
CuCl_2_/MWCNT	25	/	690	38	/	200 °C	/	(58)
MnCl_2_/MWCNT	25	/	590	33	/	200 °C	/	(58)
MgCl_2_/MWCNT	25	/	550	20	/	200 °C	/	(58)
CaCl_2_/MWCNT	25	/	470	18	/	200 °C	/	(58)
MWCNT	25	/	120	85	/	/	/	(58)
NaBr/GA	20	7.4	690	4	75	80 °C	stable capacity over 20 cycles	(28)
CaCl_2_/COF (TAPT-DMTA)	25	1	450.5	240	34	80 °C, vacuum 2 h	/	(63)
SrCl_2_/Sr-zeolite X	22	3	306.17	95	45	/	capacity maintained at over 92% after 10 cycles after removing the detaching SrCl_2_	(52)
SrCl_2_/Sr-zeolite A	22	3	149.6	257	21	/	/	(52)
Sr-zeolite A	22	3	121.55	371	0	/	/	(52)
Sr-zeolite X	22	3	160.48	390	0	/	/	(52)
Ca-zeolite A	22	3	142.63	432	0	/	/	(52)
Na-zeolite X	22	3	176.12	473	0	/	/	(52)

### 2.1 Porous Materials for Ammonia Adsorption

Porous materials such as MOFs and COFs are state-of-the-art materials, emerging in various fields, especially gas separation. MOFs exhibit large surface area, well-defined frameworks, and can form myriad topological structures and chemical components because they are comprised of metal clusters and organic ligands. Lately, MOFs have attracted attention due to their high designability, resulting from the adjustability of surface chemical activity (functional groups, hydrophobicity) and tunable pore properties (pore surface area, pore volume, size, and shape).^30^ MOFs and COFs interact with ammonia through weak physisorption interaction. The pore size can be adjusted to be comparable to the size of the NH_3_ molecule to enhance physical adsorption. Furthermore, postsynthetic modification of MOFs can also enhance the chemical adsorption of NH_3_ molecules. Various functional types are reported, especially open metal sites, acidic type, hydrogen bonding, and dipole–dipole interactions.^31^ High ammonia adsorption capacities were reported under STP conditions. A significant number of MOFs with open metal sites have been studied, such as Cu_2_Cl_2_BBTA,^32^ Mg_2_(dobpdc),^33^ Co(NA)_2_,^34^ Fe-soc-MOF,^35^ NU-1000-Cl-120,^36^ with ammonia capacities of 336 mg/g, 406 mg/g, 298 mg/g, 250 mg/g, and 180 mg/g, respectively. Ammonia can also be adsorbed via acidic sites, as in the case of Ni_acryl_TMA,^37^ Ga-PMOF^38^ with NH_3_ capacities of 400 mg/g and 179 mg/g, respectively. Another possible interaction with NH_3_ is through hydrogen bonding, as in the case of MIL-101,^39^ and MOF-303(Al)^40^ with capacities of 170 mg/g and 335 mg/g, respectively. All reported capacities are at 25 °C and 100 kPa. Unfortunately, these materials are suitable for NH_3_ storage at standard temperature and pressure, but the challenge for MOF implementation for ammonia separation remains their negligible adsorption capacity and low stability at high temperatures. Current research and development priorities include ligand functionalization, the use of transition metals as components in MOFs, and safer storage and release.

### 2.2 Metal Halides for Ammonia Absorption

**Figure 1:**
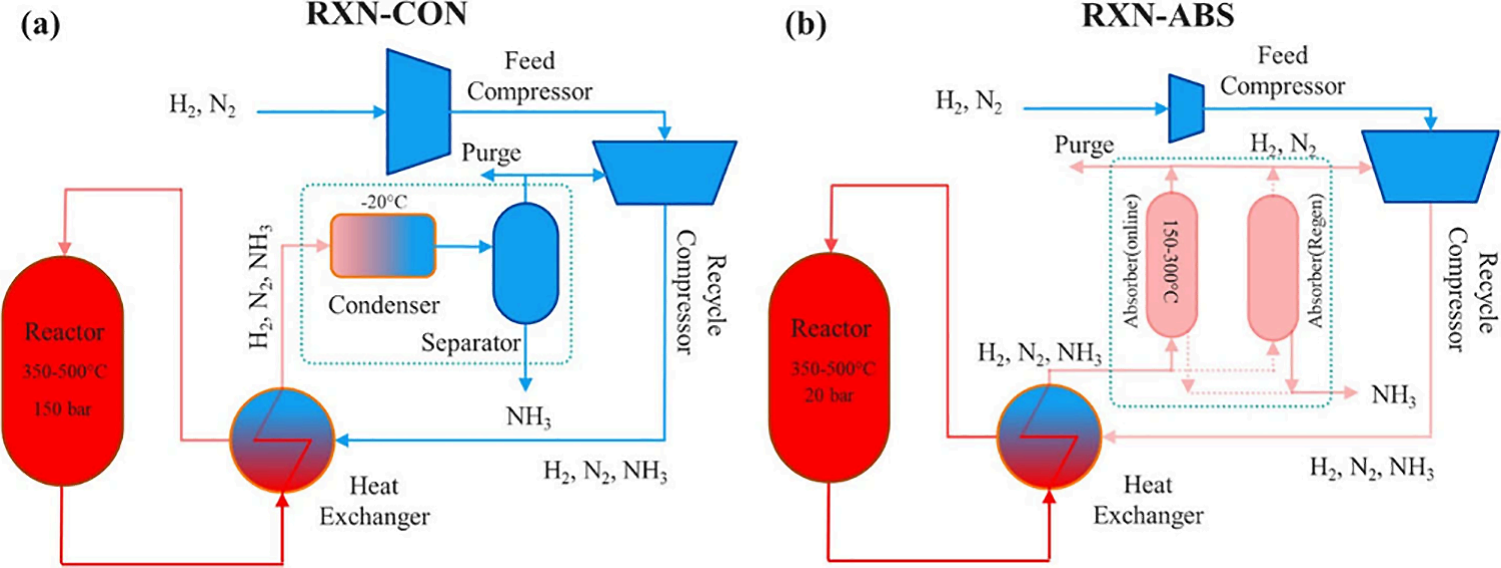
Ammonia synthesis loop block flow diagrams for (a) reaction-condensation in a conventional HB process and (b) reaction-absorption process where the condenser is replaced by absorber columns. Reprinted with permission from ref (43). Copyright 2020 American Chemical Society.

A strong interest in these compounds was aroused, in particular, by the development of ligand field theory and chemical heat pumps. The latter were first proposed by Faraday, who introduced the idea of a cooling device, based on an NH_3_–AgCl working pair in 1824.^41^ Ammonia-based heat pumps are of great interest because of their effective, low-grade thermal utility, cost efficiency, and environmental friendliness. However, the conformational instability of metal halides during the sorption process still stands in the way of their applicability.^28^ Lately, metal halides have also been proposed as ammonia-absorbing materials in the Haber–Bosch process instead of the conventional condensation process as presented in Figure 1. When sorbents are used, the ammonia produced by the catalyst is removed from the system, which in turn prevents the backward reaction and turns the equilibrium toward ammonia production according to Le Chatelier’s Principle.^42^

The ammonia molecule has a nonbonded electron pair and is therefore a Lewis base that can form coordinate covalent bonds with a Lewis acid, such as metal ion.^44^ Metal halides can form metal-ammonia complexes with different coordination numbers. A great advantage of metal halides as ammonia separation materials is reversibility, which enables a convenient ammonia recharge process.^45^ Metal halides are the most promising among inorganic ammonia sorbents because of their remarkable absorption capacity compared to zeolites, silica, and MOFs. MgCl_2_, CaCl_2_, SrCl_2_, MnCl_2_, and bromine analogues have been extensively studied,^19,46^ with MgCl_2_ having the highest ammonia capacity of about 1001 mg/g at room temperature and 1 bar among bulk halides (Table 1).^47^ CuCl_2_, CuBr_2_, and CuI have also been proposed as ammonia-absorbing materials, with CuCl_2_ and CuBr_2_ forming hexammines, but CuI not forming ammines, probably due to the low binding force of cuprous iodide to ammonia.^48^ Furthermore, the absorption behavior of various earth metal halides, including MgCl_2_, CaCl_2_, CaBr_2_, SrCl_2_, and SrBr_2_, and their hydrated forms has been studied at 25 to 200 °C. The ammonia absorption capacity of MgCl(OH) was 444 mg/g, which is approximately 5.5 times higher than that of Na-exchanged Y zeolite.^49^

#### 2.2.1 Supported Metal Halides

The efficient use of metal halides continues to present some challenges, particularly due to the structural changes that occur upon ammonia absorption. Halides are not always stable during multiple absorption–desorption cycles, as they can undergo a volume change of about 400%, which in turn leads to structural disintegration.^28,52^ For example, in the case of copper halides, the absorption capacity decreased by 40% in the second cycle compared to the first cycle and then remained almost the same until the tenth cycle. A significant decrease in specific surface area and pore volume was observed, which was attributed to sintering.^48^ The agglomeration of particles becomes more obvious with the increase in temperature, showing the phenomenon of bulk sintering, which involves changes in the microstructure of the material and results in decreased number of mesopores.^51^ The diffraction patterns of Ba_0.5_Sr_0.5_Cl_2_ before and after seven ammonia cycles showed good stability, although a broadening of the reflection peaks was observed due to the fragmentation of the crystallites as a result of the lattice strain caused by the volume changes during the first ammonia absorption. The crystallinity is reduced after several cycles.^53^ Because the voids are present after desorption, particles easily segregate with each other. The process of agglomeration changes the structure and reduces both the cyclic performance and the mass transfer kinetics during ammonia resorption.^54^ To avoid such problems and improve the performance of bulk metal halides, they are often dispersed on porous supports with large surface area, intrinsic structure and pore properties,^46,55^ e.g. on activated carbon (AC), zeolites, siliceous materials, alumina, expanded natural graphite (ENG), carbon nanotubes (CNTs) and MOFs.^19,28,56^ When the support is used, it works as a framework between the metal halide particles, limiting the agglomeration process.^51^

The sorption mechanisms for ammonia are different for zeolites than for alkaline earth metal halides (AEMHs). Zeolites are physisorbents and adsorb ammonia through weak interactions, whereas AEMHs strongly absorb ammonia by forming coordination complexes. Therefore, the ammonia desorption energies for zeolites are usually lower than 40 kJ/mol, while desorption energies for AEMHs are higher than 40 kJ/mol. Because of the lower energy of ammonia release, the rapid kinetics of gas sorption in zeolites, and their chemical stability, composites are a potential solution to overcome the limitations of using bulk AEMHs.^52^ In this way, diffusion of ammonia into the dispersed metal halide clusters occurs more rapidly and may not limit ammonia uptake.^46,55^ In general, supported metal halides exhibit excellent capacities near their thermodynamic equilibrium limits, significantly outperforming the capacities of unsupported analogues.^19^ Composite materials also exhibit faster kinetics than bulk salts. The porous matrix can accommodate the swelling of the salt during the reaction between ammonia and the salt. Therefore, it is important to select a suitable host matrix.^19,56^

##### Zeolite Supports

Microporous materials such as zeolites have been extensively studied for gas sorption applications because they possess high specific surface area. Especially alkaline earth chlorides and bromides on silica and zeolite Y (FAU) are currently considered the most promising materials for ammonia removal.^19^ Compared to bulk CuCl_2_, MnCl_2_, MgCl_2_, and CaCl_2_, silicon-supported analogues showed significantly higher NH_3_ sorption capacity. For instance, the capacity of MnCl_2_ on silicon support was approximately 128% higher than that of pure MnCl_2_. When silicon was used as the supporting framework, the capacity was improved because the particles did not agglomerate.^51^ Cao et al.^52^ used zeolite A and zeolite X as supports for calcium and strontium salts, as both possess high chemical and thermal stability. At 22 °C, SrCl_2_-loaded zeolite X and NaCl_2_-loaded zeolite X reportedly had the highest sorption capacities of 306 mg/g and 176 mg/g, respectively. Hollow mesoporous silica spheres (HMSS) were also used as the support for MgCl_2_. At 30 °C, an ammonia capacity of 340 mg/g was observed for 80 wt % loading, exceeding the sorption capacity of the pure HMSS matrix by 4.7 times. Most of the MgCl_2_ entered the middle channel of the HMSS, and a small fraction remained on the surface and in the mesoporous channel. The impregnation of the HMSS support with metal halide can ensure good dispersion and accessibility of ammonia, prevent the agglomeration of pure MgCl_2_, and thus increase the ammonia sorption capacity.^57^ The functionalization of the mesoporous material with metal halide can significantly improve the sorption performance, since such a composite material has both physical and chemical sites to improve the ammonia capacity. Consequently, the support adsorbs ammonia, while the metal halide simultaneously absorbs it.^57,58^

##### 2.2.1.2 Carbon Additives

Carbon materials such as graphite, graphene, and carbon nanotubes have been proposed as excellent additives for metal halides to improve their performance as ammonia absorbents, not only reducing agglomeration but also improving their permeability by increasing thermal conductivity.^47,59−61^ AC loaded with MgCl_2_ has shown promise as a sorbent, potentially applicable in industrial pressure swing adsorption (PSA) processes for hydrogen extraction, for both ammonia removal and enrichment. A 4 wt % Mg in MgCl_2_-AC maintained its high sorption capacity after 15 cycles, but also performed well in breakthrough experiments. The ammonia capacity was 146 mg/g at 40 °C and 7 bar, which is approximately 93% higher than that of AC alone.^62^ Cao et al.^47^ studied graphite (Gt) and graphene nanoplatelet aggregate (GNA)-MgCl_2_ composites prepared by ball milling. The composites exhibited rapid ammonia sorption kinetics in the low temperature regime, which was attributed to the increased surface area and microporosity. MgCl_2_-GNA and MgCl_2_-Gt had sorption capacities of 810 mg/g and 795 mg/g, respectively, at 25 °C. The carbon additives provide the structural stability even at high temperatures. The increase in surface area and porosity in GNA provides additional channels for the ammonia gas and reduces the agglomeration of MgCl_2_. The additional channels serve as diffusion pathways to enhance mass transfer during ammonia sorption and increase the gas permeability of the composites. Compared with pristine multiwalled carbon nanotubes (MWCNTs), the ammonia capacity of several metal halides and MWCNTs composites was enhanced, although the specific surface area and porosity were significantly reduced after the sorption cycles.^58^ Cao and Akhtar reported much faster sorption kinetics of the structured porous SrCl_2_-rGO (reduced graphene oxide) composite compared to that of pure SrCl_2_ pellet. The fast kinetics is the result of the increased surface area provided by the pores of the rGO networks. Its excellent elasticity and the voids in the structure allow the material to adapt to the volume expansion of SrCl_2_ during the sorption process by self-adjusting “breathing,” thus maintaining the macro- and microstructure,^54^ while the SrCl_2_ particles in the pellet form segregated. Additionally, the properties of the composite, such as porosity, pore size, and loading, can be readily adjusted by changing the parameters in the freeze-casting synthesis method, e.g., freezing rate and suspension concentration.^54^

##### 2.2.1.3 Other Supports

A number of hybrid materials have been investigated to capture ammonia. By confining CaCl_2_ in a COF, a high capacity of 450.5 mg/g was reported at 25 °C and 1 bar. The efficiency of the sorbent is the result of a high dispersion of the halide in the pores of the COF due to strong host–guest interactions, but also a coordinating interaction between ammonia and Ca^2+^ in combination with hydrogen bonding between ammonia and Cl^–^. COFs not only have high crystallinity and a large surface area, but also a highly tunable structure and functionality.^63^

**Figure 2:**
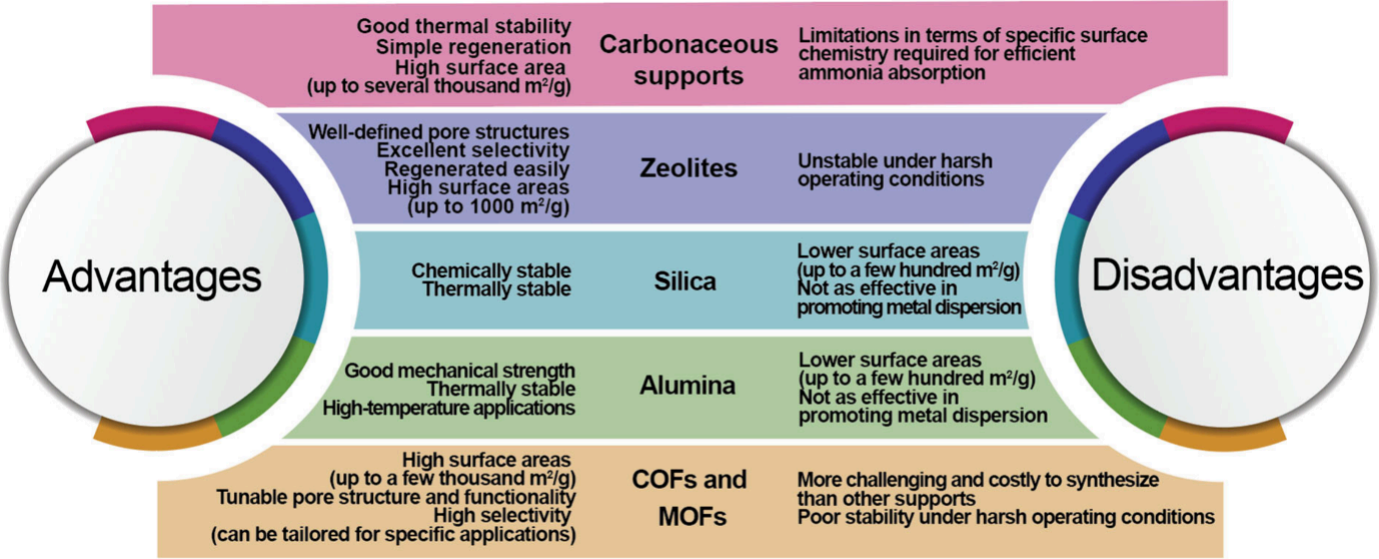
A comparison of advantages and disadvantages of different supports used for metal halides as ammonia sorbents.

**Figure 3:**
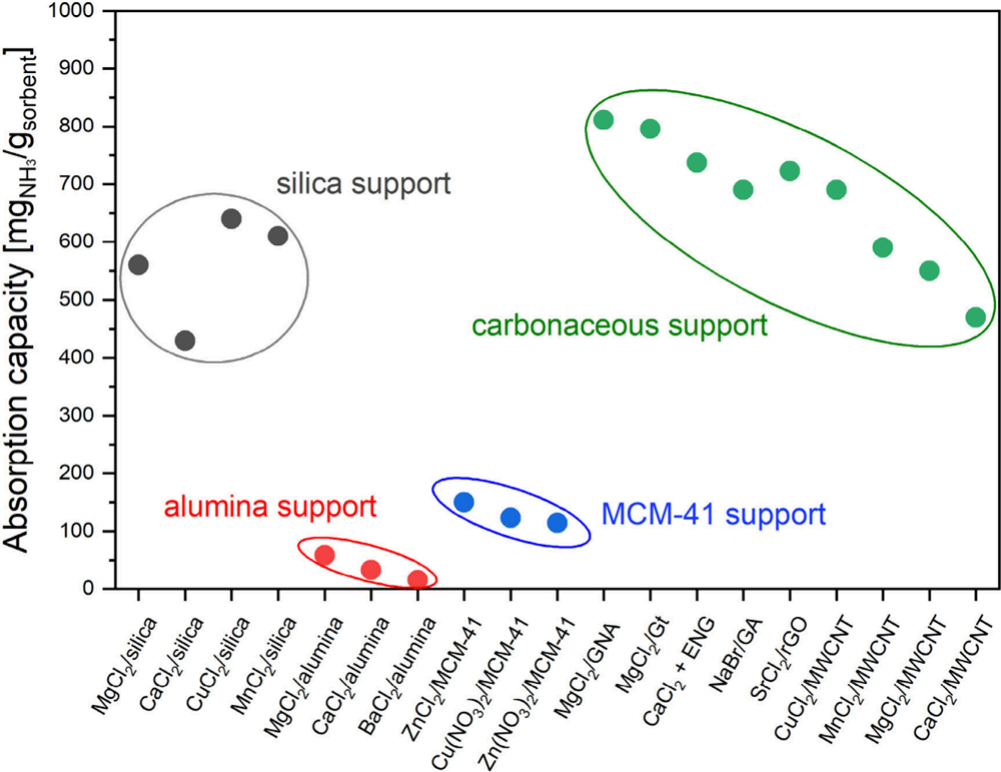
Ammonia absorption capacity of different materials at 25 °C taken from Table 2.

The optimal choice of the support requires careful consideration as it depends on several factors, e.g. surface area requirements, mechanical strength, thermal stability, chemical compatibility, regenerability and cost. In terms of surface area, activated carbon, zeolites and certain MOFs are preferred. High surface area materials improve the dispersion of metal halides and increase the active sites available for ammonia sorption. For processes requiring high temperatures, supports with excellent thermal stability, such as silica, alumina or certain zeolites, are preferred as they also allow sufficient regeneration. From the comparison of the ammonia capacities of supported and unsupported metal halides, it appears that the ammonia sorption of composite sorbents mainly depends on the chemical absorption of NH_3_ by metal halides (Table 2).^58^ Silica and carbonaceous materials currently appear to be the most promising among the different supports for metal halides (Figures 2 and 3). The mechanism of ammonia sorption of the composite sorbents at low temperatures is a combination of physical adsorption and chemical absorption, with chemical absorption predominating for metal halides.^58^

#### 2.2.2 Binary and Ternary Systems

**Figure 4:**
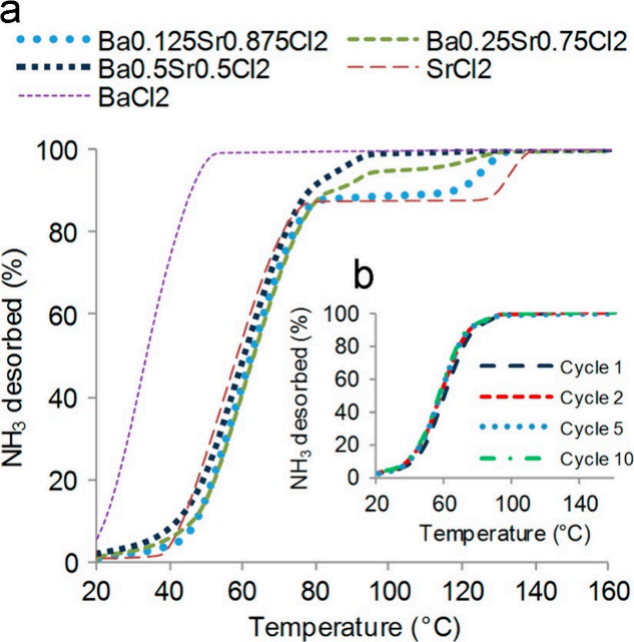
Ammonia temperature-programmed desorption (TPD) curves. (a) Ammonia TPD from Ba_*x*_Sr_(1–x)_Cl_2_ (*x* = 0.125, 0.25, and 0.5). (b) Ammonia TPD from Ba_0.5_Sr_0.5_Cl_2_ after 1, 2, 5, and 10 saturation cycles. Reprinted with permission from ref (53). Copyright 2014 Elsevier.

A few studies have been performed on mixed metal halide (MMH) sorbents. The results for mixed cations (MgCaBr, MgSrBr, CaSrBr) show that their absorption capacities are almost equal to the average values for pure salts or at least between the values for pure salts. Similar results were obtained for mixed anions (CaClBr, MgClBr, and SrClBr), indicating that the materials studied are mixtures of pure salt crystals rather than crystals containing an actual mixture of earth metals or halides.^19^ The equilibrium pressure curve for Ca_0.5_Sr_0.5_Cl_2_ was intermediate between those of Ca(NH_3_)_8_Cl_2_ and Sr(NH_3_)_8_Cl_2_.^68^ Similarly, Liu and Aika investigated the NH_3_ absorption behavior of various MMHs and reported that halide mixtures with a common anion resulted in separate phases of the halide components and did not mix as solid solutions. In contrast, mixtures with a common cation formed solid solutions.^69^Figure 4 shows that pure Sr(NH_3_)_8_Cl_2_ releases 7 NH_3_ molecules below 80 °C, while the last molecule is desorbed above 125 °C. In pure Ba(NH_3_)_8_Cl_2_, all 8 NH_3_ molecules are desorbed below 50 °C. The introduction of barium into Sr(NH_3_)_8_Cl_2_ leads to a desorption pattern for the first 7 molecules that is very similar to the desorption from pure Sr(NH_3_)_8_Cl_2_. The main effect of increasing barium concentration is a shift in the desorption of the last ammonia molecule toward lower temperatures. The mixed salts also exhibit better properties than the two individual components in terms of usable gravimetric and volumetric ammonia densities.^53^

Berdiyeva et al.^70^ investigated the thermodynamic properties of the mixed metal halide Mg_0.5_Mn_0.5_(NH_3_)_6_Cl_2_ and found that desorption occurs in three steps, with activation energies significantly lower than those of the desorption steps of Mg(NH_3_)_6_Cl_2_. A binary halide was also stable in the temperature range of 20–350 °C and upon ammonia cycling. The NH_3_ release temperatures of the MMH ammines Mg_1–*x*_Mn_*x*_(NH_3_)_6_Cl_2_ (*x* = 0–1) were between those of the pure metal halide ammines. The absorption kinetics of Mg_0.5_Mn_0.5_Cl_2_ was shown to be similar to that of MnCl_2_, indicating that Mn plays a predominant role in determining the kinetics of hexammine formation. Depending on the application, the NH_3_ desorption temperature and kinetics of the investigated stable metal halide solid solutions can be adjusted by changing the relative Mg/Mn ratio. A first high capacity ternary metal halide (Ba_4_CaSr_3_Cl_16_) was synthesized by Jensen et al. The ternary halide was reported to behave slightly differently from the binary Ba/Sr metal chlorides, as it showed release in a broader interval, which could be the result of slower kinetics. However, the stability was similar to the binary metal halide ammines, as almost no changes in working capacity were observed.^71^

## 3 Influence of Different Parameters on Absorption

Absorption capacity depends on several parameters and can be varied considerably, by changing salt chemistry, metal halide loading, and physical conditions of absorption. To effectively separate and purify ammonia in a cyclic absorption-based process, the absorption/desorption conditions, such as pressure, temperature, and purge gas must be carefully optimized.^29^

### 3.1 Salt Type and Metal Oxidation State

Among the halides, chlorine (Cl) and bromine (Br), are the most common anions due to their low molar mass and industrial maturity.^72^ Although bromides display a higher capacity than chlorides, the difference is not significant enough to justify using bromides instead of the generally cheaper and more stable chlorides.^19^ Among cations, alkaline earth metals are most commonly used, especially Mg^2+^, Ca^2+^, and Sr^2+^.^73^ Cations with smaller atomic numbers generally show greater affinity for ammonia and form more stable ammines, which is indicated by decreasing stability down through the groups for the cations. The alkali metals show decreasing stability in the order Li > Na, and the stability of the alkaline earth metals decreases in the order Mg > Ca > Sr. The same trend is observed in the transition metal groups, where stability decreases in the order Ni > Co > Fe > Mn, Cu > Ag > Au, and Zn > Cd > Hg.^74^ Be forms covalent bonds with Cl instead of ionic bonds, Ba^2+^ is toxic, and Ra is radioactive, while K and Rb do not form metal ammine halides that would be stable at room temperature, so these elements are rarely found in the literature.^73^ Several studies have shown that magnesium salts have higher ammonia capacities than calcium salts, while both absorb more ammonia than strontium salts.^19,29^ The dynamic sorption capacity increases in the row BaCl_2_ < CaCl_2_ < MgCl_2_ < MnCl_2_ < CuCl_2_.^51,75^ The type of metal has a much greater effect on ammonia capacity than the oxidation state of the cation. Zinc, copper, and iron nitrates, chlorides, sulfates, and carbonates supported on MCM-41 were thoroughly investigated and it was concluded that the type of metal and the type of anion significantly affect ammonia the capacity, while the pH and the oxidation state of the metal have no effect on the ammonia capacity.^64^

### 3.2 Metal Halide Loading

According to several studies, the metal salt content has a great influence on the sorption performance of ammonia. Furtado et al.^64^ observed a volcano shaped dependence of ammonia capacity on loading, with the highest ammonia capacity of about 150 mg/g when loaded with 50 wt % ZnCl_2_ at room temperature exceeding the capacity of pure MCM-41 by more than four times. As the loading increases, the sorption capacity initially increases, but when the loaded amount exceeds a certain threshold, the ammonia capacity decreases.^57,62,65^ Lower loadings have lower capacities because there are fewer accessible metal sites for reaction with ammonia. Contrariwise, larger loadings can result in salt fusion, obscuring metal sites and offering less surface area for diffusion into the salt.^19^ The salt is present in concentrations too high to be effectively dispersed throughout the porous matrix, resulting in clogging of the mesoporous channels of the support as well as clumping of the salt and reduced surface area for chemisorption.^57,64^ The optimum salt loading on the support is thus between 40 and 50 wt %. The masses of salt and support should be approximately equal^19^ so that small salt crystals are prevented from fusing by the support matrix surrounding them.^50^ The amount of metal loaded onto the support is critical for optimizing the pore properties, which are a crucial parameter for high sorption performance.^62^

### 3.3 Ammonia Absorption at Higher Temperatures

**Figure 5:**
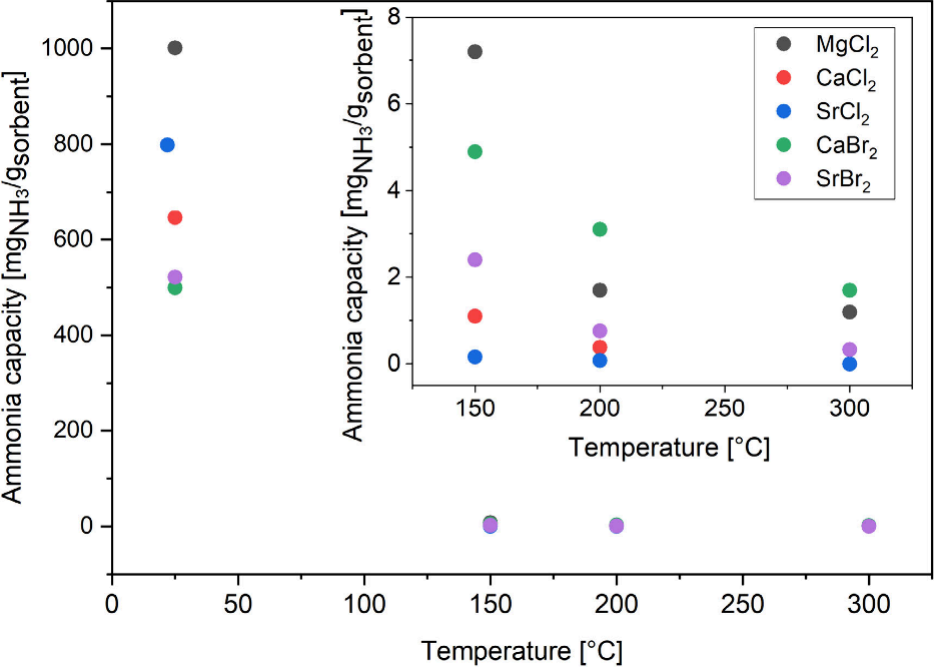
Ammonia absorption dependence on temperature of absorption for bulk metal halides at low pressures (for values at 25 °C at 1 bar, and for higher temperatures at 4 bar). For a better visual representation of capacities at higher temperature, an insert plot is added in the temperature range of 125 to 325 °C.

Although there are a variety of materials that are excellent for removing ammonia from gaseous streams at room temperature, the release of ammonia from these materials begins at temperatures as low as 40 °C. Furthermore, cyclic ammonia uptake and release from most materials can cause the materials to decompose, changing their mesoporous or microporous structure and thus reducing ammonia capacity. Almost none of these materials can effectively remove ammonia at temperatures above 200 °C. Both the initial and working capacities of the sorbent decrease as the absorption temperature increases, as can be seen from Figure 5 for bulk metal halides, because a lower temperature is thermodynamically more favorable for ammonia absorption.^19,29,62,76^ The desorption rate increases at higher temperatures because more thermal energy is available. In contrast, absorption rate decreases with increasing temperature.^67^ Because absorption is much more selective than adsorption, metal halides have a greater high-temperature capacity than MOFs or zeolites.^27^ However, the results indicate that metal halides in their bulk form are not suitable as high-temperature ammonia absorption materials as their ammonia absorption capacity does not exceed 8 mg/g. What also makes them inefficient is their low stability–the breakthrough of the subsequent cycle decreases after each absorption/desorption cycle.^19^

Of the various porous supports, silica and zeolite Y showed the best results at higher temperatures, while alumina, kaolinite, and diatomaceous earth had low apparent capacity.^19^ Malmali et al.^19^ performed a comprehensive study of sorbents at temperatures used in existing ammonia synthesis and obtained ammonia capacities of up to 70 mg/g at 150 °C and 4 bar for MgCl_2_ and CaCl_2_ loaded at 40 wt % on silica. Hrtus et al.^29^ showed that sorption capacity of silica loaded with MgCl_2_ is 200 mg/g at 25 °C, which is significantly higher than at temperatures above 200 °C, where the capacity is approximately 60 mg/g. Further, Shen et al.^51^ investigated the ammonia sorption performance of metal chlorides both in bulk and on silicon supports. The sorption capacity of the metal halides decreases and the agglomeration of the particles becomes more evident at higher temperatures, revealing the phenomenon of bulk sintering. When silicon was used as the supporting framework, the capacity improved, because the particles could not clump together.

It is important to emphasize that the equilibrium capacity of metal halide must be the same regardless of the type of support and whether the metal halide is supported or not. Different capacities are therefore not the result of changes in the diffusion coefficient of ammonia into the salt, but are rather due to the fact that the surface area per mass of salt changes due to the distribution of the salt on the support. Therefore, supports with large BET surface areas, such as silica and zeolite Y, perform better in sorption tests. However, this is not the rule for alumina.^19^ For instance, the capacity of MgCl_2_ on alumina is 58.6 mg/g at 25 °C and 11.6 mg/g at 300 °C.^41^ Therefore, other factors such as surface tension must also play a role.^19^ Wagner et al.^50^ reported that MgCl_2_ on alumina support still has a capacity almost three times higher than pure MgCl_2_ at 150 °C. The supported sorbent showed reproducible performance over many cycles, which can be attributed to the fact that the MgCl_2_ crystals are confined in similarly sized pores of the alumina, preventing microstructure degradation during cycling. MgCl_2_ in bulk, on the other hand, loses capacity over time, because of fusing and deterioration of microstructure. After exposure to ammonia at high temperature, metal halides form a single dense solid mass, which means that fewer interfaces are available for absorption.

### 3.4 Balancing Temperature and Pressure

Pressure and temperature are the two most important factors in the ammonia sorption process, so pressure-swing adsorption (PSA) and temperature-swing adsorption (TSA) are two common gas separation processes. In the PSA process, the adsorbents/absorbents are regenerated by reducing the pressure, whereas in the TSA process, regeneration is achieved by applying heat.^65^ While a higher temperature favors the reaction rate, a lower temperature favors thermodynamic equilibrium.

The increase in pressure enhances adsorption capacity due to an increased number of gas molecules colliding with the surface of the adsorbent, which raises the probability of gas molecules adhering to the surface.^77^ In porous materials, higher pressure can force more gas molecules into the pores, filling up more available space and thereby increasing the overall adsorption capacity.^50^ According to the Langmuir isotherm, adsorption capacity increases with pressure until a saturation point. Furthermore, increased pressure enhances the driving force for mass transfer, resulting in higher flux through the material. Similarly to adsorption, absorption capacity also increases by the increase in pressure, due to the increased likelihood of interactions between the gas molecules and the absorbent material, leading to more gas molecules being absorbed. For processes involving a chemical reaction, chemical equilibrium also plays a crucial role. Increasing the pressure can shift the equilibrium toward the formation of the absorbed species, thereby increasing absorption capacity.^62^ Moreover, increasing the total pressure raises the partial pressure of each gas component, resulting in a higher driving force for each gas to dissolve or react with the absorbent material.

All in all, although pressure thus affects both thermodynamics, as well as dynamics (transport, kinetics···) of ad-/absorption, it is ordinarily much more likely that the number of surface active sites (e.g., halides) is more determining, should an order of magnitude be concerned. Hrtus et al.^29^ found that combining TSA with PSA significantly improves the performance of the metal halide absorbent, also achieving ammonia purity greater than 90%. PSA was able to increase the working capacity of the sorbent by 1.6 times even at high temperatures (300/400 abs/des). The best cyclic adsorption capacities are achieved when absorption temperatures are lowered and desorption temperatures are increased.

## 4 Metal Halide Structure and Mechanism of Ammonia Absorption

**Figure 6:**
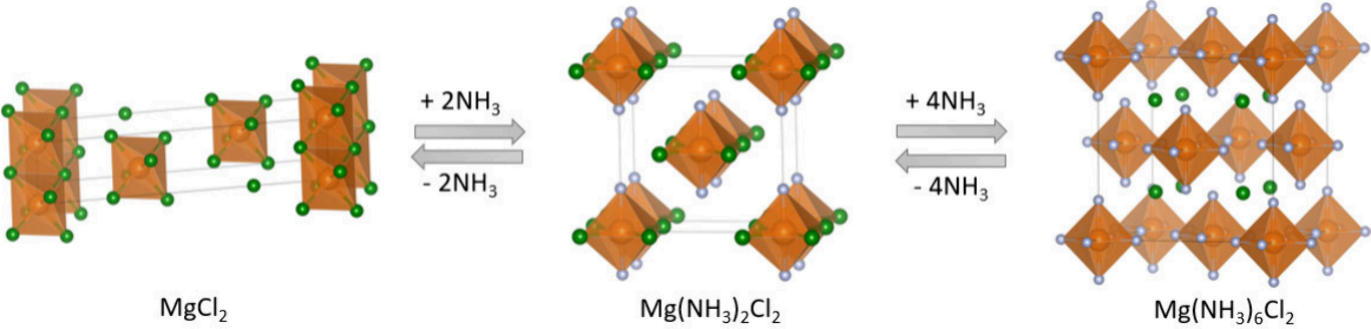
Crystal structures of (a) MgCl_2_ (PDF No. 04-008-7748), (b) Mg(NH_3_)_2_Cl_2_ (PDF No. 04-009-8931), and (c) Mg(NH_3_)_6_Cl_2_ (PDF No. 04-010-3690) with space groups *R*3̅*m*, *Cmmm*, and *Fm*3̅*m*, respectively. Color scheme: Mg, orange; N, gray; Cl, green; H is omitted for clarity. The structure of Mg(NH_3_)Cl_2_ is not presented as the CIF file could not be obtained.

Ammonia reacts with metal halides to form a metal coordination complex, which is accompanied by a change in crystal structure. In general, the reaction can be written as
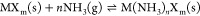
R1where M denotes a metal and X denotes halides. Taking the commonly used MgCl_2_ as an example, the reaction with ammonia proceeds in three steps as follows:^65^
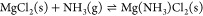
R2
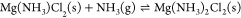
R3
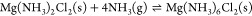
R4In the structure, a central magnesium cation is coordinated octahedrally to six ligands *n* (chlorine atoms for *n* = 0, ammonia molecules for *n* = 6, or the combination of both) as shown in Figure 6. The possible coordination of ammonia ligands for some metal halides along with the space group symmetries is shown in Table 3. Magnesium halide and 3d-transition divalent metal halides tend to form hexammines and can reach high gravimetric densities of ammonia, whereas strontium, calcium and barium chlorides can react with ammonia to form octammines.^65^

**Table 3:** NH_3_ Absorption Number, Space Group, Crystal System, and Corresponding Enthalpy and Entropy of Metal Halide Ammines Reported in Literature^a^

material	absorption no.	space group	crystal system, Bravais lattice	Δ*H* [kJ/mol]	Δ*S* [J/(mol·K)]
MgCl_2_	0	*P*3̅*m*1^71^	Trigonal	H_6–2_ = 55.6 (43)^82^	H_2–6_ = 230.6^49^
		*R*3̅*m*^81^	Trigonal	H_2–1_ = 74.9 (52)^82^	H_1–2_ = 230.3^49^
	1	unknown	unknown	H_1–0_ = 87.0 (81)^82^	H_0–1_ = 230.9^49^
	2	*Cmmm*(83)	Orthorhombic		
	6	*Fm*3̅*m*^71^	Cubic		
SrCl_2_	0	*Fm*3̅*m*^84^	Cubic	H_8–2_ = 43.4 (36.0)^85^	S_8–2_ = 235.6 (253.2)^85^
	1	*Cmcm*(85)	Orthorhombic	H_2–1_ = 58.9 (49.4)^85^	S_2–1_ = 270.1 (265.1)^85^
	2	Aem2^85^	Orthorhombic	H_1–0_ = 45.4 (48.1)^85^	S_1–0_=/ (235.3)^85^
	8	*Pnma*(85)	Orthorhombic		
CaCl_2_	0	*P*4_2_/*mnm*^71^	Tetragonal	H_4–8_ = 41.0^49^	S_4–8_ = 230.3^49^
	1	unknown	unknown	H_2–4_ = 42.3^49^	S_2–4_ = 229.9^49^
	2	Ccme^71^	Orthorhombic	H_1–2_ = 63.2^49^	S_1–2_ = 237.3^49^
	4	unknown	unknown	H_0–1_ = 69.1^49^	S_0–1_ = 234.1^49^
	8	*Pnma*(53)	Orthorhombic		
MnCl_2_	0	*R*3̅*m*^70^	Trigonal	H_6–2_ = 47.3^86^	S_6–2_ = 148.5^86^
	1	unknown	unknown	H_2–1_ = 71.1^86^	S_2–1_ = 153.6^86^
	2	*Cmmm*(86)	Orthorhombic	H_1–0_ = 84.1^86^	S_1–0_ = 154.0^86^
	6	*Fm*3̅*m*^86^	Cubic		
NiCl_2_	0	*R*3̅*m*^87^	Trigonal	H_6–2_ = 47.4^78^	/
	1	I2/m^88^	Monoclinic	H_6–2_ = 71.0^78^	
	2	*Cmmm*(89)	Orthorhombic	H_1–0_ = 84.2^78^	
	6	*Fm*3̅*m*^90^	Cubic		
Mg_0.5_Mn_0.5_Cl_2_	0	*R*3̅*m*^70^	Trigonal	/	/
	1	I2/m^70^	Monoclinic		
	2	*Cmmm*(70)	Orthorhombic		
	6	*Fm*3̅*m*^70^	Cubic		

a The values in brackets are computationally obtained.

**Figure 7:**
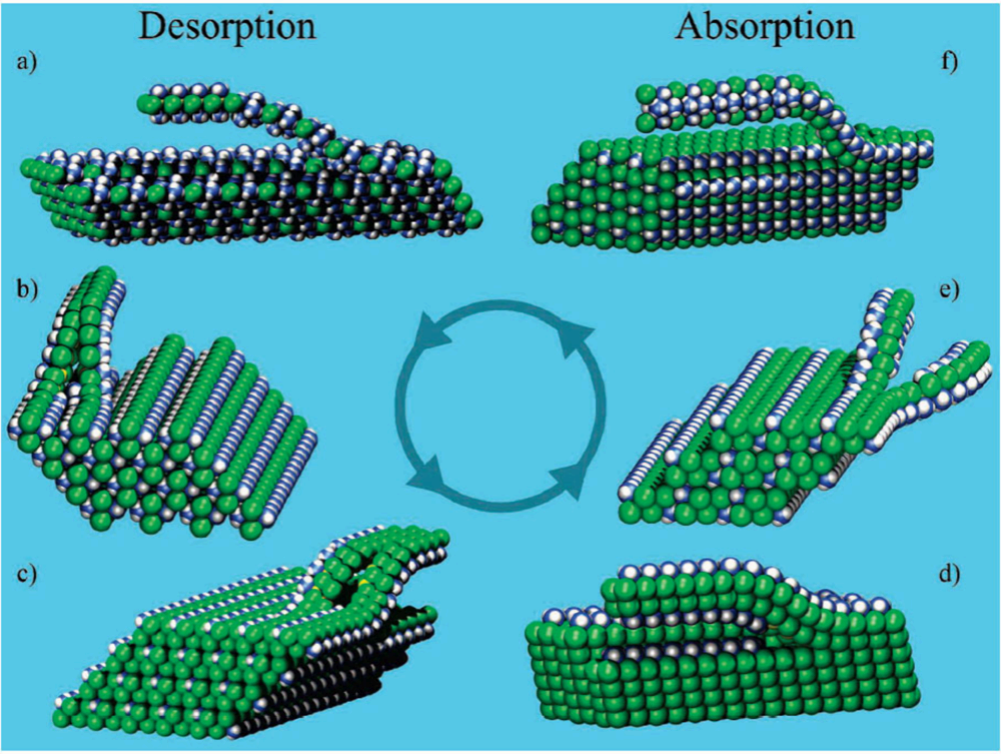
Proposed mechanism for absorption/desorption of ammonia in Mg(NH_3_)_*n*_Cl_2_, Mn(NH_3_)_*n*_Cl_2_, and Ni(NH_3_)_*n*_Cl_2_. Panels (a–f) depict structures in one absorption/desorption process. Reprinted with permission from ref (78). Copyright 2008 American Chemical Society.

Bulk MgCl_2_ has a layered structure, while *n* = 1, 2, or 6 ammine structures consist of chains. Sørensen and co-workers set up a density functional theory (DFT) model to explain the fast absorption–desorption mechanism for M(NH_3_)_*n*_Cl_2_ (M = Mg, Mn, Ni) ammines by applying a model of chain abstraction from the surface. In this model, NH_3_ molecules are proposed to enter and leave the bulk interface via zipping/unzipping of the loosely bound chains of octahedral ML6 groups, where M is the metal and L is the ligand (either NH_3_ or Cl, depending on the number of NH_3_ molecules absorbed), to allow the conservation of metal coordination geometry independent of *n*. The model also includes a structural rearrangement in the bulk after each successive sorption step (Figure 7). During the absorption/desorption process, the material transitions from one phase to another such that the layers or chains of the original structure are cleaved/recombined to absorb/desorb ammonia.^78^

A simple microkinetic model based on the adsorbed state was developed by Ammitzbøll et al.,^79^ supported by DFT calculations. The ammonia molecules are first adsorbed on the surface of the material (Langmuir adsorption, eq 1), then cross the diffusion barrier to the bulk sites, near the surface, and subsequently diffuse deeper into the material. The mass balance between the molecules at the surface and the gas molecules was used (eq 5). The model does not account for transport constraints, such as bulk diffusion within the crystals, because the time scale to reach equilibrium between the gas phase and the surface is several orders of magnitude faster than all other time scales. For SrCl_2_, *ΔE*_*in*_ = 10 kJ/mol is the barrier that an ammonia molecule must overcome when moving from the surface to the bulk (eq 2–4).
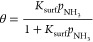
1
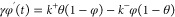
2
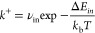
3
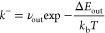
4
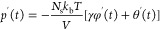
5where θ is the surface coverage, *K*_surf_ is the equilibrium constant resulting from the van’t Hoff relation. φ is the bulk site occupancy, and γ is the ratio between the number of bulk sites and surface sites. *N*_s_ is the number of surface sites, and *V* is the gas volume in the reactor. Physical parameters of the reactor and the binding energy of the adsorbed state from the thermogravimetric analysis were used in the model.^79^ Pan et al.^57^ studied supported MgCl_2_ on HMSS and the results show that NH_3_ molecules are first absorbed on the outer surface of the sorbent at relatively low pressure. Then, the molecules gradually penetrate into the inner surface of the sorbent at relatively high pressure, resulting in an increase in absorption capacity. If the support has a uniform pore size, the ammonia gas can easily penetrate into its inner pores, where it coordinates with the metal halide in the pore. In this way, the capacity is further increased by physical and chemical sorption. Interestingly, Aoki et al.^80^ demonstrated that at room temperature, the hexammine complex Mg(NH_3_)_6_Cl_2_ is directly formed by the reaction between MgCl_2_ and NH_3_. Thermodynamically, mono- and diammine complexes are more stable, but the formation of low-coordinated ammine complexes is prevented by the high kinetic barrier, which is probably related to symmetry–hexammine has a more symmetrical structure as seen in Table 3. Given the scarcity of established kinetic and microkinetic models, the precise absorption mechanism remains elusive. To attain a thorough comprehension of the underlying sorption process, it is imperative to conduct additional computational investigations alongside experimental validations.

## 5 Kinetic Modeling

For a variety of metal halide applications, including chemical heat pumps, ammonia separation, ammonia storage and delivery as well as selective catalytic reduction of NOx gases, absorption/desorption dynamics are critical to performance. Effective kinetic models have been studied in this context.^79^ Coordination chemistry materials generally release ammonia in multiple steps that are, in the case of metal halide-supported salts, kinetically limited.^29^ There are several types of kinetic models, either phenomenological or analogical, that are fundamentally different. Phenomenological models require a comprehensive analysis of the reacting medium, with kinetics comprised of various elementary mass transfer and purely chemical reaction mechanisms. These complex models require a profound understanding of the physicochemical properties of the reacting medium. Analogical approach, in the contrast, aims to take into consideration all the phenomena in a global manner. Instead of including elementary reaction mechanisms, the analogical model attempts to reproduce their overall effect, also considering the medium as an equivalent entity. The kinetics becomes independent of the physicochemical properties of the medium. Although analogical models are only valid under the conditions, used to obtain the parameters, they are well adapted to simulation in situations of geometric similarity in the case of scale changes and are easier to develop.^91^ Usually, kinetic models that account for sorption, describe the reaction rate in terms of:
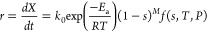
6where *X* is the degree of conversion of the reaction, *k*_0_ and *M* are constants, *E*_a_ is the activation energy, *R* is the gas constant, *T* is the absolute temperature, and *s* is the degree of saturation of the ammonia.^79,91^ Several different forms of the function *f* have been studied, most commonly a linear form *f = P*_rel_(*s,T*), a power form *f = P*_rel_(*s,T*)^*N*^, and a logarithmic form *f =* log(*P/P*_*e*q_). Here, *P* is the pressure, *N* is another constant, and *P*_rel_ is a relative pressure given by the following relation:
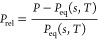
7

Unlike adsorption, absorption involves a chemical reaction. Therefore, the kinetics can be described by an equilibrium constant according to the van’t Hoff relation, which is simplified to an equilibrium pressure of ammonia (*P*_eq_), since it is the only gas involved in the reaction. If an excess ammonia concentration is present, absorption is complete above the equilibrium pressure.^76^*P*_eq_ is calculated using van’t Hoff equation:
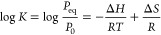
8*ΔH* and *ΔS* are the enthalpy and entropy changes, respectively, *K* is the equilibrium constant, and *P*_0_ is the reference pressure. The kinetic parameters are usually divided into three groups. Pseudo-orders of reaction (*M*) affect the way reactivity changes as the reaction proceeds. The kinetic coefficients (*k*_0_) reflect the evolution of the physical conditions in the medium. Finally, the pseudoenergies of activation (*E*_a_) determine the sensitivity of the kinetics to temperature.^91^ To obtain the most accurate results, each parameter should be determined independently.

Kubota et al.^46^ used the grain model in the power form to analyze the kinetics of absorption/desorption of ammonia in unsupported metal halides. Assuming the presence of a reaction intermediate, the rate expression is as follows:
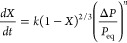
9where *P* is the pressure of NH_3_ in Pa, and *ΔP = P – P*_eq_ or *ΔP = P*_eq_ – *P* for absorption or desorption, respectively. The pressure index term, *n*, represents the magnitude of the dependence of the rate on the ammonia pressure, and *k* is the reaction rate constant [1/s] of the reaction studied. *n* and *k* were determined by thermogravimetric experiments at different reaction temperatures and partial pressures. The ammonia partial pressure was found to have a greater effect on desorption than on absorption. The activation energies in the system were determined using the Arrhenius plot.

Lebrun and Spinner^91^ used an analogical model in the logarithmic form for the calcium chloride-methylamine pair. Kinetic and thermal parameters were determined by daisy-chaining sequences together, with the results of each stage serving as a starting point for the following one. Ammitzbøll et al.^79^ obtained the best fit to experimental data with the linear form *f*, but observed a deviation from the effective kinetic model at pressures approaching equilibrium pressure, suggesting that there is an adsorbed state in the absorption dynamics. A simple microkinetic model based on the adsorbed state, mentioned above, was also implemented. Unlike the kinetic model, the microkinetic model was consistent with the data.^79^ Assuming that ammonia first interacts with the supported halide through physical adsorption before absorption occurs, Smith and Torrente-Murciano^76^ modeled the kinetics of ammonia separation as a three-step process, with the first term describing adsorption and the second and third terms describing absorption in the following equation:
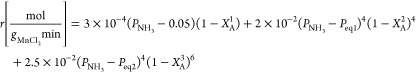
10The equation includes the difference in ammonia pressure, the fraction of capacity achieved (*X*_A_^*k*^) in each step (*k*) due to ammonia accumulation in the sorbent. The adsorption step is described in the linear form, while the absorption steps are in the power form. Though the initial rate of ammonia removal is in accordance with the experimental data, it deviates slightly with time, which is probably the result of the highly inhomogeneous structure of the thickly loaded salt. Therefore, a pinpoint determination of such dynamic sorption process is unlikely. After subtracting the adsorption term, the same kinetic model was also used for MnCl_2_ in bulk, which confirmed the significant decrease in surface area.^76^

Wagner et al.^50^ proposed a simplified model of Weber and Chakravorti^92^ to model the performance of a fixed-bed adsorption column, with the bed divided into 20 equal units. The model was based on the assumptions that alumina is impregnated with MgCl_2_ only in the outermost layer, the surface of the support is not completely covered, and the particles are homogeneous. Therefore, diffusion into the particles of the support does not affect absorption into the metal halide. Experimental data were used to include the capacity of alumina only when necessary, since alumina is not expected to adsorb ammonia at higher temperatures and lower partial pressures. Two concentrations, one for the outside of the particles (*C*_1_) and one for the interior of the particle (*C*_2_), were modeled:
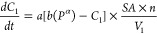
11
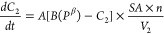
12where *A* and *a* are rate constants in μm/s, while *b* and *B* are partition coefficients in mol/[μm·bar^α(β)^]. α and β are dimensionless constants to quantify the effect of pressure, and *n* is a multiplication factor to include porosity. In the proposed model, the absorption rate was proportional to the square root of time, indicating that absorption is diffusion-driven.^50^ The desorption rate is more affected by reaction temperature than the absorption rate.^46^ There is still much debate about how to best model the mechanism of absorption.^76^ The kinetic models are also important because they can be integrated into the computational fluid dynamics (CFD) model, which can also provide a visual understanding of species distributions within the reactor, allowing optimization of reactor design and operation.^93^

## 6 Thermodynamics

**Figure 8:**
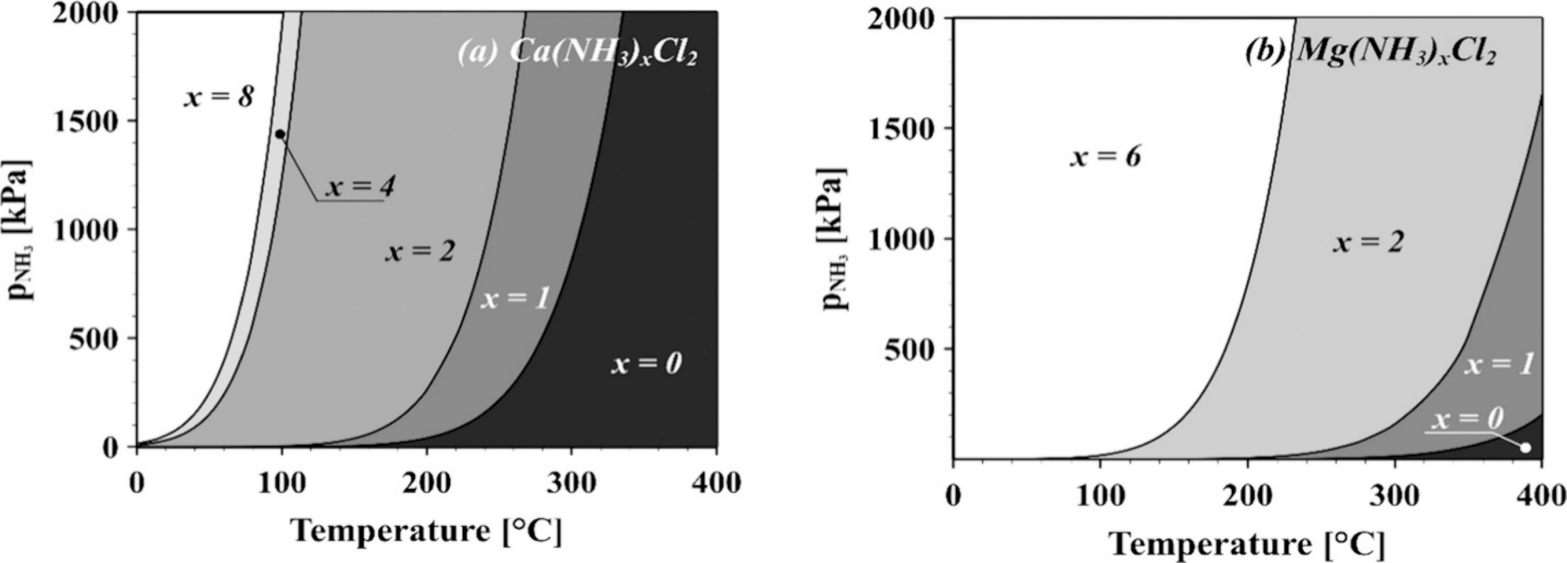
Temperature–pressure–composition diagrams of (a) ammoniated CaCl_2_ and (b) ammoniated MgCl_2_ showing their phases with the corresponding coordination numbers. Reprinted with permission from ref (29). Copyright 2022 American Chemical Society.

Absorption is usually a multistep reaction in which the steps approximate to molar equivalents of the absorbent salt.^76^ Evaluation of the thermodynamic properties of ammonia sorption on halide salts can provide important data for ammonia storage, thermal engineering, and indirect hydrogen storage applications.^94^ Aoki et al.^95^ reported the correlation between plateau pressure and electronegativity of cation/anion forming metal halides. Materials with higher Pauling electronegativity (χ_p_) of the cation exhibited lower plateau pressures, while the materials with higher electronegativity of the anions exhibited higher plateau pressures. Consequently, metal halides with a lower electronegativity difference between the cation and anion have much lower equilibrium pressures.^95^ Furthermore, absorption involves a chemical reaction, so the thermodynamic properties such as enthalpy change (*ΔH*^0^) and the entropy change (*ΔS*^0^) for the specific reaction can be evaluated by the van’t Hoff equation (in the literature also referred to as the Clausius–Clapeyron relation):^76,95^
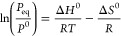
13when the plateau (equilibrium) pressure at the experimental temperature is known.^95^ Hrtus et al.^29^ used the van’t Hoff equation to obtain a temperature–pressure composition diagram (Figure 8). Such diagrams can be used to determine whether complete desorption is possible under the desired conditions.

Liu and Aika^49^ investigated the absorption behavior of several metal halides and reported that *ΔS* was almost constant in the range of 210–240 kJ mol^–1^ K^–1^, while *ΔH* differ significantly for different cation and anion species as well as for the number of coordinated ammonia molecules.^49^ Metal halide ammines are formed in an exothermic reaction between a dry salt and ammonia. Although ammonia uptake is often observed at ambient conditions, some halides such as LiF, KBr, MgF_2_, CaF_2_, NaCl do not form ammine complexes at ambient conditions.^95^ Desorption, conversely, is an endothermic, usually multistep reversible process in which desorption temperatures vary considerably for different materials.^96^ For most hexammines, a three-step thermal release of ammonia occurs. At an ammonia backpressure of 1 bar, Mg(NH_3_)_6_Cl_2_ first releases 4 NH_3_ molecules at 142 °C, then another molecule at 230 °C, while the last molecule is released at 375 °C and the MgCl_2_ is regenerated. The same three-step release is observed for Mn(NH_3_)_6_Cl_2_, where the observed release temperatures are 80 °C, 180 °C, and 354 °C for the release of 4 NH_3_, 1 NH_3_, and 1 NH_3_ molecule, respectively. For Ni(NH_3_)_6_Cl_2_, the corresponding desorption temperatures are 168 °C, 327 °C, and 396 °C.^46,97^ The charge density of the metal cation is related to the desorption temperatures. Cations with a higher charge-density bind ammonia more strongly, which is also reflected in the ionic radii for Mg^2+^ (0.72 Å), Mn^2+^ (0.83 Å), and Ni^2+^ (0.69 Å) for coordination 6.^98^

Although the chemisorption reaction was previously considered reversible, metal halide-ammonia working pairs exhibit desorption hysteresis phenomenon. For this reason, it is challenging to determine the overall sorption characteristics, meaning both absorption and desorption, by performing measurements on only one of the two.^94,95^ There are several theories regarding the phenomenon. According to Zhong et al.^99^ the reason could be the temperature difference between the measured gas phase and that inside the salt particle. In addition, it could also be related to the expansion and contraction of the crystal lattice as the ammonia content in the metal halide-ammonia complex changes. Thus, the hysteresis may be caused by changes in the solid phase, with the different phases having different crystal structures. During absorption, some energy is consumed due to volume expansion and rearrangement of atoms, whereas during the desorption process, these atoms undergo free contraction without energy release.^99,100^ Recently, Wu et al.^94^ performed measurements in the thermal quasi-equilibrium state, where the temperature lag could be ignored. Therefore, it is more likely that the hysteresis is the result of the expansion and contraction of the crystal lattice. Gao et al.^101^ reported a significantly decreased hysteresis behavior for multi salt sorbents. So far, it is still unknown why multisalt sorbents with certain compositions can weaken hysteresis phenomenon.

## 7 Computational Methods for Prediction of Novel Materials for NH_3_ Separation

The synthesis of new materials, especially mixed or doped compounds, is frequently guided based on chemical knowledge and intuition. Although materials can be continuously improved through experimental research, synthesis and characterization can be extremely time-consuming because of a large number of parameters being altered and testing of all compounds is practically impossible. Furthermore, material properties are usually only slightly modified. Computational techniques, particularly screening studies, can be used to systematically test a wide variety of combinations by replacing elements in the structure. With increasing role of artificial intelligence (AI), particularly genetic algorithm (GA) and machine learning (ML), computer-assisted screening has recently gained prominence as it can not only screen out unstable compounds, or the ones unsuitable for a particular application, but are also faster and cheaper, and do not produce chemical waste.^71,102^ Because the investigated search space often contains thousands of candidates, the detailed examination of materials would be computationally demanding. Hence, screening based on template structures is often an effective tool, involving calculations on structures in known crystal symmetries.^71^

DFT calculations are often used to study crystal structures, determine thermodynamic properties of the materials, such as the desorption enthalpies of the studied systems as well to propose a mechanism for the better understanding of rapid absorption/desorption processes.^71,103^ In DFT calculations, it is important to select an appropriate exchange-correlation (XC) energy functional. In studies of metal halide ammines, the computationally more demanding van der Waal’s (vdW-DF) corrected functional is used because it accounts for the dispersion and van der Waal’s forces, which are crucial for the description of metal halide ammines because they contain a large number of hydrogen bonds between the ammonia molecules and the halides.

In 2007, Sorensen et al.^78^ used DFT calculations to accurately reproduce the trends in desorption enthalpies of Mg(NH_3_)_6_Cl_2_, Ca(NH_3_)_8_Cl_2_, Mn(NH_3_)_6_Cl_2_, and Ni(NH_3_)_6_Cl_2_. A mechanism of rapid ammonia absorption/desorption has been proposed in which individual chains of the ammines are released from the crystal surface. It was reported that desorption from metal ammine salts is not limited by either diffusion kinetics or large activation energies, but rather by thermodynamic equilibrium (heat transport to the reaction zone).^78^ DFT was applied alongside quasielastic neutron scattering by Tekin et al.^82^ to study the crystal structures of Mg(NH_3_)_*n*_Cl_2_ (*n* = 1, 2, 6), bulk diffusion, and rotation kinetics of NH_3_ through the corresponding transition state(s). The activation barrier calculations showed a good agreement with the experimental enthalpies. Reportedly, the release of NH_3_ is limited by bulk diffusion, so lowering these barriers would improve the overall kinetics of the system. Hydrogen bonds between the hydrogen atom of NH_3_ and the chlorine atoms of the halide play a crucial role in stabilizing the metal-ammine complex. The group of Lysgaard et al.^85^ combined desorption measurements, XRPD, and DFT to identify thermodynamically stable phases of strontium ammine halides. The crystal structures of Sr(NH_3_)Cl_2_, Sr(NH_3_)_2_Cl_2_, and Sr(NH_3_)_8_Cl_2_ were solved in the *Cmcm*, Aem2, and *Pnma* space groups, respectively. Compared to the monoamine, the diamine phase was found to have marginally higher or lower stability, depending on temperature and pressure, which is why this phase is not found in many experiments. By using DFT it was possible to determine the reaction enthalpy of the transition from the di- to monoammine.^85^ Johnsen et al.^104^ studied structural transformations in the crystal structure of the Sr(NH_3_)_8_Cl_2_ complex as a function of temperature and pressure of the ammonia gas. During the absorption/desorption process, the crystal structure changes significantly. Here, DFT calculations were used to obtain an information on the energy required to alter metal–nitrogen bond length.

*Ab initio* calculations were also applied by Yamane et al.^105^ to gain insight into the microscopic properties of NH_3_ absorption by metal chlorides (MnCl_2_) and metal borohydrides (MnBH_4_), particularly the effect of cations and anions on sorption. Additionally, the molecular dynamics (MD) simulations were performed under the canonical ensemble (N, V, T) and the temperature was controlled with the Nosé thermostat (300 K) to study the stability of the system. The NH_3_ absorption properties are mainly governed by NH_3_-cation interactions, as the absorption energy decreases in the order Li > Na > K and Mg > Ca, and much less of NH_3_-anion repulsion.^105^ Bialy et al.^53^ obtained a series of new stable barium strontium chloride solid solutions by advanced computational material prediction combined with experimental data. Various metal ratios were investigated and DFT calculations were performed to predict the structural properties and thermodynamics of MMH ammines, and the results were in excellent quantitative agreement with experimental results.

**Figure 9:**
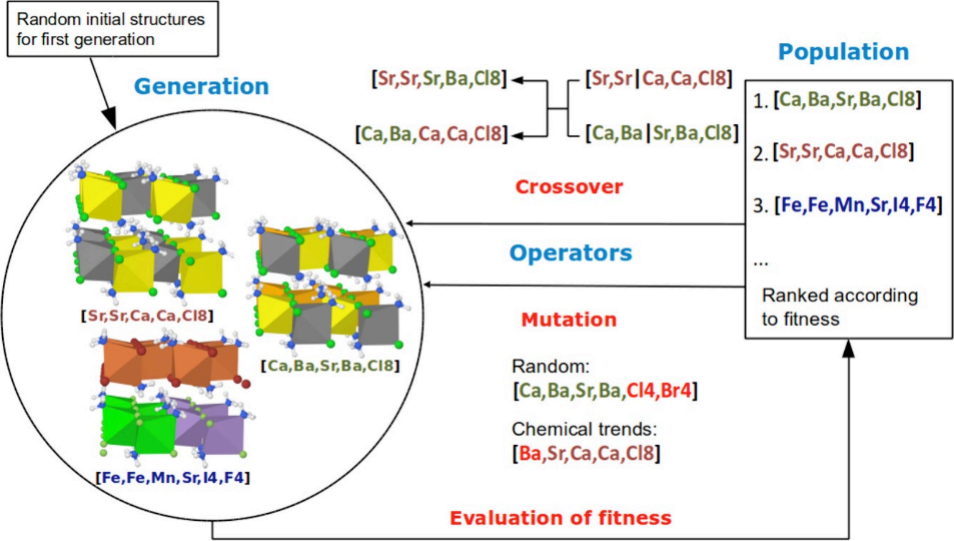
Genetic algorithm illustration searching for optimized material mixtures using templates. The individual candidates are encoded as vectors describing the contained metals and their position in the templates. Using various operators, the algorithm generates new trial candidates by using the information from one or more parents. Reprinted with permission from ref (108). Copyright 2016 Hydrogen Energy Publications LLC.

DFT calculations guided by GA, also commonly used for other classes of materials,^106,107^ are extremely useful in identifying new mixed metal halide chlorides because the screening of the defined search space often involves a very large number of different structures. The operators, of which crossover and mutations are most often applied to generate offspring, are a crucial component of a successful GA (Figure 9).^71^ Jensen et al.^102^ predicted new mixed metal hexammines with hydrogen storage capacities that may release ammonia in one step over a predefined temperature interval. Several stable candidates were reported, including Ti_2_CuMgCl_8_, Ti_2_ScVCl_8_, Ti_3_VCl_8_, and ScCu_2_MgCl_8_, with predicted release temperatures. Not surprisingly, these structures are rich in titanium, as it is among the lightest elements in the search space.^102^ In another study, new stable mixed metal halide chlorides were investigated for ammonia storage. Twenty-four selected metals, including the alkaline-earths and the 3d and 4d transition metals with up to three different metals in the structure, were allowed in the screening, resulting in 100,000 different structures in the defined search space. Among the potential materials for ammonia storage, a high-capacity ternary metal halide ammine (Ba_4_CaSr_3_Cl_16_) was identified and subsequently synthesized.^71^

In recent years, the integration of DFT calculations with ML techniques has emerged as a promising approach in finding novel materials with preferable properties. This combination leverages the accuracy of DFT with the computational efficiency of ML, allowing for the mitigation of their respective limitations.^109,110^ Instead of the traditional high-throughput computing, combining the methods can significantly reduce the amount of first-principles calculations and save computing resources on account of expanding the range of candidates through predictive screening of ML.^109^

Training data for ML can be obtained from published literature, open databases in material science, or high-throughput computations.^111^ Performing DFT calculations on smaller set of materials provides a highly accurate training data set with key sorption properties, namely binding energies, surface properties and electronic properties, used to train a ML model. Trained ML models allow for rapid, efficient screening of new materials with desired sorption characteristics across vast chemical spaces. DFT can subsequently be used again for the validation or refinement of ML predictions.^109,112^ The combined DFT and ML approach has recently been used by de Rezende et al.,^113^ for the efficient and accurate prediction of deammoniation reaction energies for MMHs comprised of Mg, Ca, Cl, and Br. The integrated theoretical and experimental approach is clearly beneficial and effective for predicting and designing new materials for physical and chemical modifications, e.g., by changing crystal structures and bond lengths. The approach using ML provides a large number of material possibilities through reliable and fast predictions of material properties, and can among other applications be used in gas separation, catalysis and energy storage.^112,113^

## 8 Integrated Ammonia Synthesis–Separation Process

Several studies have shown that it is possible to overlap the temperature range for catalytic activity and that for ammonia sorption, so that the integrated synthesis-separation system is able to achieve conversions beyond equilibrium.^76^ Low-temperature catalysts (<300 °C), usually based on ruthenium, are preferred for the integrated process, while the sorbent must be able to retain ammonia at relatively high temperatures aligned with the synthesis. MgCl_2_ and CaCl_2_ are two conventional absorbents for ammonia separation because they are abundant. Unfortunately, CaCl_2_ is not the best option for integrated processes because it does not absorb ammonia at reasonable partial pressure above 300 °C. CaCl_2_ can decompose in the presence of water vapor, but the reaction is very slow and requires weeks of exposure to atmospheric moisture.^29^ MgCl_2_, on the other hand, can retain ammonia up to temperatures approaching 400 °C, but can absorb water from the air and decomposes to magnesium oxide and hydrogen chloride when heated above 300 °C. Precautions are therefore required to eliminate any moisture in the nitrogen and hydrogen feed gases.^114^ Since an ammonia synthesis catalyst is much more sensitive to water vapor, no traces of water should be present in the gas system anyway.^29^

**Figure 10:**
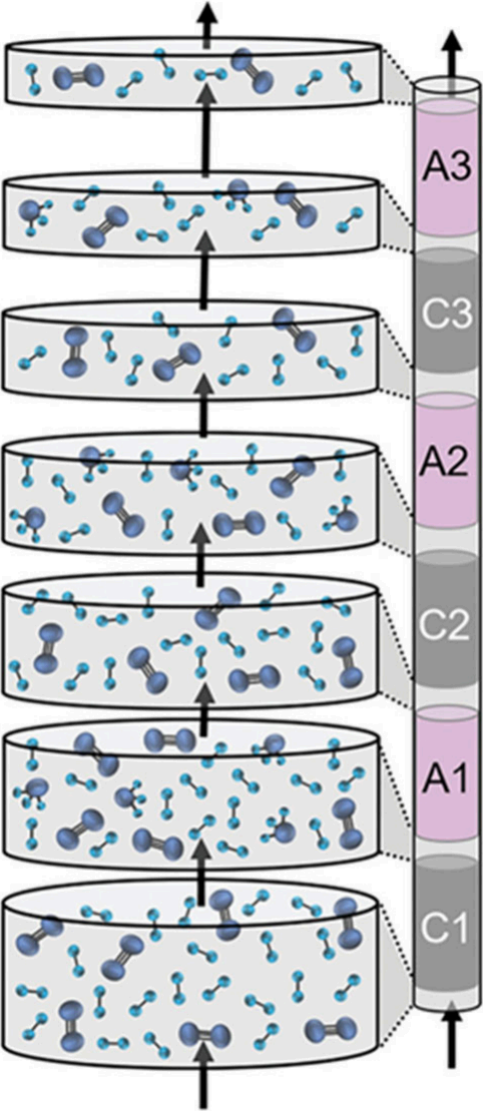
Representation of an integrated catalyst–absorbent flow system with 1:1 volume ratio. Scheme is depicting a decrease in flow after every layer of catalyst (CX) and absorbent (AX), where X represents the layer number. Reprinted with permission from ref (76). Copyright 2021 John Wiley and Sons.

Smith and Torrente-Murciano^76^ used a 5%Ru/10%Cs/CeO_2_ as catalyst and MnCl_2_ on a SiO_2_ support as sorbent in the sorption separation process (Figure 10) in which the overall hydrogen conversion of >90% was achieved. Unlike the two salts mentioned above, MnCl_2_ releases water below 200 °C and is thus stable at higher temperatures. Malmali et al.^115^ investigated a reaction separation process at reduced operating pressure, where the sorptive separation of ammonia was nevertheless achieved by CaCl_2_. Reportedly, CaCl_2_ is an appropriate absorbent for the removal of ammonia, even at temperatures close to the reaction temperature.

Currently, several methods of field-assisted ammonia synthesis, including photocatalysis, nonthermal plasma catalysis, and electrocatalysis, offer an attractive prospect for operation under mild conditions. Electrochemical ammonia synthesis offers the possibility to reduce N_2_ directly from water and air to NH_3_ under ambient conditions. Both reactants can be produced in the same cell as two opposing half-reactions.^116,117^ Photochemical synthesis enables the use of abundant N_2_ and H_2_O as feedstocks under mild conditions and without carbon emission.^118,119^ Plasma-assisted synthesis offers clean, sustainable, adaptable, carbon-free ammonia production at low temperatures and atmospheric pressure and is suitable for on-site production, using plasmas to activate the feed gases and drive the process.^120,121^ Such processes for NH_3_ production based on solar or wind energy would be an attractive prospect as part of an integrated ammonia synthesis–sorption system operating at low temperatures.^118,122,123^ While these environmentally friendly synthesis routes offer the potential for integration with sorbents, they face certain challenges. These include difficulties in N_2_ dissociation and low selectivity in electrochemical and photochemical synthesis, as well as inefficiencies in conversion and effectiveness in the case of plasma-assisted synthesis.^116,119^ Recently, nonthermal plasma-assisted ammonia production has been proposed as a prominent alternative to the currently used production. Peng et al.^114^ studied the mechanism of catalytic absorption, using MgCl_2_ as the absorption material. Interestingly, the absorption proceeded via two pathways in which Mg_3_N_2_ or Mg(NH_3_)_6_Cl_2_ was formed. The Mg_3_N_2_ intermediate formed via the nitridation mechanism under plasma conditions. The absorbent was placed at different positions with respect to the plasma discharge region. The best results in terms of plasma efficiency, ammonia synthesis and absorption rate were obtained when the absorbent was in the plasma discharge region. It was reported that MgCl_2_ provided not only the absorption but also the dielectric effect.^114^ When a conventional condenser process is replaced by an absorber, the increase in production rate per catalyst mass is limited to approximately 10%. If the recycling rate is simultaneously increased, the increase in the production rate per gram of catalyst can even exceed 1000%.^27^

In the design and optimization of an integrated synthesis-sorption process, mathematical modeling of the kinetics is of great use.^76^ Experimental analysis as well as model predictions show that both reaction and separation rates are fast, and therefore the recycle flow is the rate-limiting step in the proposed integrated process.^115^ Although models identify important parameters for integrated system design, many still do not include regeneration of the sorbent by increasing the temperature, decreasing the pressure, or both, which is critical for integration system.^76^

## 9 Challenges and Perspectives

A wide range of materials have been proposed as media for ammonia separation, with particular attention given to metal halides, due to their ability to form strong coordination complexes with ammonia. Their major advantage is low cost and the ability to separate ammonia at high temperatures with high selectivity. The separation is sharp, the regeneration of the metal halides is simple, and the kinetics often fast. However, a major challenge is their high sensitivity to moisture and structural stability issues caused by volume swings, which can lead to a loss of efficiency. This can be overcome by supporting the halides on porous supports. There are still many limitations associated with the sorption–based ammonia synthesis process, particularly regarding sorbent stability over absorption/desorption cycles, optimization of the integrated synthesis–sorption reactor designs, lower capacity at elevated temperatures, exploration of sorption kinetics, and the development of effective kinetic models. The main research focus areas in future directions on ammonia absorption by metal halides for enhancing the energy efficiency for the separation, storage, or removal of ammonia include:

- Experimental investigation of new composite sorbents, their thermodynamic properties and ammonia sorption performance to enhance their performance, especially at higher temperatures.
- Understanding of the fundamental ammonia-metal halide interaction mechanisms to predict and control the sorption process, and examination of the influence of moisture on ammonia sorption.
- Development, scale-up and testing of ammonia sorption materials in integrated ammonia synthesis and sorption-based separation processes and investigation of their long-term cyclic stability to ensure reversibility.
- Development of efficient kinetic models for the description of the mechanism of ammonia adsorption/absorption and desorption, as slow kinetics can limit practical performance of metal halides in sorption systems.
- Combination and improvement of computational methods like DFT, GA, and ML for the efficient screening for ammonia sorption materials. This is especially important in design of mixed metal halides to tailor specific properties, and composites.

Considerable work remains to advance the practical application of NH_3_ separation technologies, with the design of separation media and the selection of experimental conditions requiring customization to meet the specific demands of each application.

## 10 Conclusions

Ammonia, an energy-dense, carbon-free fuel, is gradually emerging as a promising complementary green vector to hydrogen as the world’s technological advances seek ways to lay the foundations for a green, sustainable future. To this end, more efficient, readily deployable production of ammonia has not yet been fully implemented. New ways to improve the conventional Haber-Bosch process are currently being explored, and an integrated synthesis-separation process appears to be a prominent alternative. Although rarely studied in the literature, photocatalysis, nonthermal plasma catalysis, and electrocatalysis, which can be operated at mild conditions, could be integrated with a fast separation reactor using solid sorbents, potentially ensuring the competitiveness of the technology. Efficient catalysts that are effective at lower reaction temperatures would be beneficial for the integrated system, as lower operating temperatures would broaden the range of sorbents available for ammonia separation.

This review provides an overview of recent advances in ammonia separation technologies, with the pivotal point on metal halide materials. The structure and mechanism of ammonia separation, kinetic modeling and thermodynamics of metal halides, as well as computational methods for developing new materials for ammonia separation are described. Although new materials for ammonia removal are on the rise, challenges remain for their practical application in the industry, particularly limited cyclic stability, and high sensitivity to moisture. The reaction kinetics of halide-ammonia working pairs still receives little attention, as researchers mainly focus on experimental results and almost no new kinetic models have been developed in recent years. The main reason for this is the lack of understanding of the underlying chemisorption mechanism. Further investigation and development of kinetic models that incorporate the preadsorbed state are needed for practical application. Finally, the development of computational methods for the efficient prediction of ammonia removal materials is required. In order to develop an efficient and cost-effective ammonia technology, an integrated knowledge from different research areas, especially catalysis, materials science, computer science and chemical engineering, is of utmost importance.

## Abbreviations

AAHP: ammonia-based heat pumps
AC: activated carbon
AEMH: alkali earth metal halides
BET: Brunauer–Emmett–Teller
CFD: computational fluid dynamics
CHP: chemical heat pump
CNT: carbon nanotubes
COF: covalent organic framework
DFT: density functional theory
DES: deep-eutectic solvent
ENG: expanded natural graphite
GA: graphene aerogel
GA: genetic algorithm
GNA: graphene nanoplatelets aggregates
Gt: graphite
HB: Haber–Bosch
HMSS: hollow mesoporous silica sphere
IL: ionic liquid
MCM-41: Mobil Composition of Matter No. 41
MD: molecular dynamics
ML: machine learning
MMH: mixed metal halide
MOF: metal–organic framework
MWCNT: multiwalled carbon nanotubes
PSA: pressure swing adsorption
QENS: quasi-elastic neutron scattering
rGO: reduced graphene oxide
STP: standard temperature and pressure
TSA: temperature swing adsorption
vdW-DF: van der Waals density functional
XC: exchange-correlation
XRPD: X-ray powder diffraction
ZIF: zeolitic imidazolate framework

## Symbols

Δ*E*_in_: energy barrier
Θ: surface coverage
*K*_surf_: equilibrium constant
φ: bulk site occupancy
γ: the ratio between the number of bulk sites and surface sites
*N*_s_: number of surface sites
*V*: gas volume in the reactor
*X*: degree of conversion of the reaction
*k*_0_: kinetic coefficient
*M*: pseudo-order of reaction
*E*_*a*_: activation energy; pseudoenergies of activation
*R*: gas constant
*r*: reaction rate
*T*: absolute temperature
*s*: degree of saturation of the ammonia
*f*: function
*P*: pressure
*N*: another constant
*P*_rel_: relative pressure
*P*_eq_: equilibrium pressure
*ΔH*: enthalpy change
*ΔS*: entropy change
*K*: equilibrium constant
*P*_0_: reference pressure
*X*_A_^k^: fraction of capacity achieved
*k*: reaction step
*C*_1_: concentration outside of the particles
*C*_2_: concentration in the interior of the particle
*A*: rate constant
*a*: rate constant
*b*: partition coefficient
*B*: partition coefficient
α: dimensionless constant
β: dimensionless constant
χ_p_: Pauling electronegativity
